# Supplementary material for: Interleukin-1 receptor-induced PGE2 production controls acetylcholine-mediated cardiac dysfunction and mortality during scorpion envenomation
Source: Nat Commun. 2020 Oct 28;11:5433. doi: 10.1038/s41467-020-19232-8 (PMC7595177; doi:10.1038/s41467-020-19232-8)
Supplement: Supplementary file 1 — Supplementary Information [file 41467_2020_19232_MOESM1_ESM.pdf]

Supplementary Information

Interleukin-1 receptor-induced PGE_2 production controls acetylcholine-mediated cardiac dysfunction and mortality during scorpion envenomation

Reis MB et al.

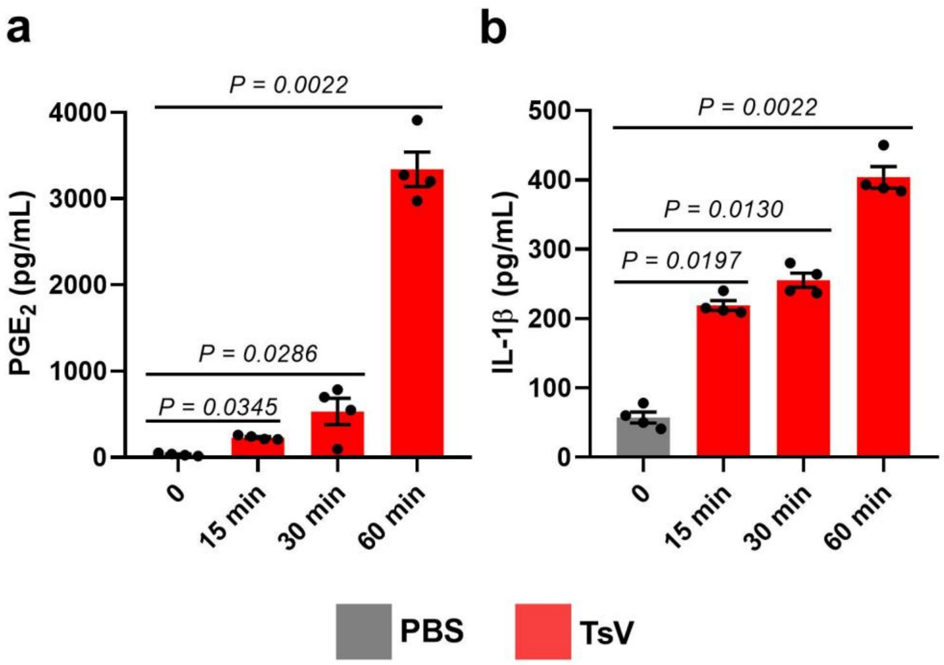

Supplementary Fig. 1 | Time-course of PGE_2 and IL-1β production in the heart of lethally-envenomed mice. (a) The concentration of PGE_2 and (**b**) IL-1β in heart homogenates from C57Bl/6 mice inoculated with PBS (300 μl/i.p.) or a lethal dose of TsV (180 μg kg^{-1} i.p./300-μl). Mice given PBS were euthanized after 60 min, and those given TsV were euthanized after 15, 30, or 60 min. Data represent means ± SEM, n = 4, one experiment. Differences were considered significant if $P < 0.05$ according One-way ANOVA followed by Bonferroni's multi-comparison test.

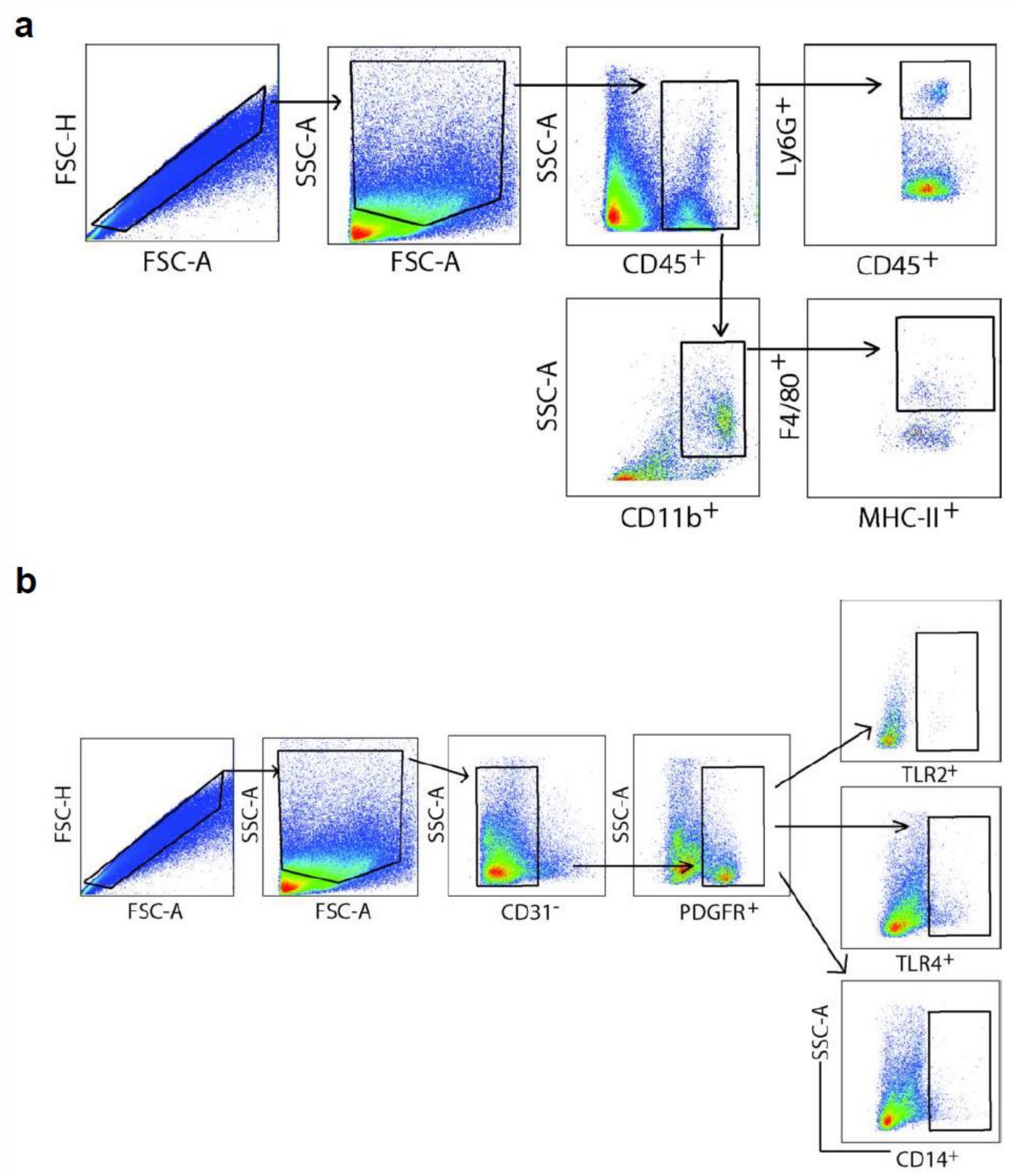

Supplementary Fig. 2 | Flow cytometry gating strategy. (**a**) Contour plots show a representative flow cytometric gating hierarchy for the analysis of FSC-H/FSC-A and SSC-A/FSC-A followed by CD45$^+$Ly6G$^+$ (neutrophils) and CD11b$^+$F4/80$^+$MHC-II$^+$ (macrophages). (**b**) Contours plots show representative flow cytometric data and gating hierarchy for the analysis of FSC-H/FSC-A and SSC-A/FSC-A followed by CD31$^-$ PDGFR$^+$ (cardiac fibroblasts) and TLR2$^+$ or TLR4$^+$ or CD14$^+$ expression.

	PBS	PBS	PBS	PBS	TsV	TsV	TsV	TsV	TsV
Alox5	0,972227	0,812091	1,168342	1,084071	0,903989	0,859917	0,968187	0,703356	1,089908
Alox5ap	1,06024	1,01915	0,93385	0,99102	0,850245	1,360195	1,160292	1,405563	1,105872
Blt1	0,52366	1,646491	0,799118	0,611209	0,643909	2,164878	0,948675	0,756179	1,381635
Blt2	0,330639	1,675843	0,584156	0,543952	0,719136	2,241064	0,844851	0,734437	1,119586
Il1r1	0,846924	0,810735	0,868852	0,845712	1,031299	1,412187	1,143609	1,016487	1,045082
Casp1	0,814193	0,829431	0,824059	0,522244	0,74559	1,339706	0,946231	1,058019	1,359883
Nlrp3	0,515463	0,599725	0,846523	0,852633	0,837941	0,888101	0,883923	1,520231	1,196406
Cd14	0,891534	1,00293	0,955863	1,046028	1,170028	2,649999	1,73888	3,25626	5,574149
Tlr2	0,703653	0,622028	0,656726	0,576122	1,072813	0,951286	0,90364	1,084351	0,790581
Tlr4	0,961001	0,894302	1,069755	0,800677	1,087697	1,035987	1,130181	1,25142	1,125438
Cd36	0,988195	0,729806	1,086511	0,722562	1,27619	1,400593	1,273476	1,154156	1,211505
Ptger2	0,612847	0,536031	0,667774	0,452719	1,255186	2,254237	1,274238	1,358129	2,254237

Supplementary Table 1. Heart gene expression of *Alox5*, *Alox5ap*, *Blt1*, *Blt2*, *Il1r1*, *Casp1*, *Nlrp3*, *Cd14*, *Tlr2*, *Tlr4*, *Cd36 and Ptger2* by qRT-PCR of PBS or TsV-inoculated mice (Fig. 1m).

	Medium	Medium	Medium	TsV	TsV	TsV
Nlrp3 6h	1,108917	-1,18006	0,071146	4,39843	4,952581	4,286999
Nlrp3 24h	-0,48412	-0,48412	-2,4894	3,874142	6,839483	6,068819
Asc 6h	-1,18465	-1,20715	-1,51007	0,448686	-0,70369	0,758464
Asc 24 h	0,678158	-0,04008	-0,63807	0,457619	2,721657	1,564466
Casp1 6h	0,212858	-1,34693	0,304813	0,832663	0,902149	1,042114
Casp1 24h	0,857865	0,300786	-1,15865	0,57184	2,747957	1,815121
Il1b 6h	0,772811	-1,74514	0,972333	11,56739	12,40688	10,77256
Il1b 24h	-1,09197	1,439241	-0,34727	17,51146	16,44201	14,95594
Ptgs2	0,6598	0,8452	0,2365	7,388411	7,782503	6,909828
Tlr2	0,857865	0,500786	0,6678	2,27011	3,25082	4,072232
Tlr4	0,695	0,257	0,977	0,388246	0,343703	0,357335
Cd14	0,795	0,653	0,946	1,995787	2,436717	2,221258
Ptger2	0,659	0,498	1,254	1,989805	3,245772	1,933423

Supplementary Table 2. Gene expression of *Ptger2*, *Ptgs2*, *Tlr2*, *Tlr4*, *Cd14*, *Il1b*, *Casp1*, *Asc*, and *Nlrp3* in CFs incubated with medium or TsV for 6 and/or 24 h (n = 3/wells per conditions).
