# Supplementary material for: Interleukin-1 receptor-induced PGE2 production controls acetylcholine-mediated cardiac dysfunction and mortality during scorpion envenomation
Source: Nat Commun. 2020 Oct 28;11:5433. doi: 10.1038/s41467-020-19232-8 (PMC7595177; doi:10.1038/s41467-020-19232-8)
Supplement: Supplementary file 3 — Reporting Summary [file 41467_2020_19232_MOESM3_ESM.pdf]

nature research

Corresponding author(s): Prof. Lúcia Helena Faccioli, PhD

Last updated by author(s): Aug 24, 2020

Reporting Summary

Nature Research wishes to improve the reproducibility of the work that we publish. This form provides structure for consistency and transparency in reporting. For further information on Nature Research policies, see our Editorial Policies and the Editorial Policy Checklist.

Statistics

For all statistical analyses, confirm that the following items are present in the figure legend, table legend, main text, or Methods section.

n/a	Confirmed	
☐	☒	The exact sample size (n) for each experimental group/condition, given as a discrete number and unit of measurement
☐	☒	A statement on whether measurements were taken from distinct samples or whether the same sample was measured repeatedly
☐	☒	The statistical test(s) used AND whether they are one- or two-sided<br>*Only common tests should be described solely by name; describe more complex techniques in the Methods section.*
☐	☒	A description of all covariates tested
☐	☒	A description of any assumptions or corrections, such as tests of normality and adjustment for multiple comparisons
☐	☒	A full description of the statistical parameters including central tendency (e.g. means) or other basic estimates (e.g. regression coefficient) AND variation (e.g. standard deviation) or associated estimates of uncertainty (e.g. confidence intervals)
☐	☒	For null hypothesis testing, the test statistic (e.g. F, t, r) with confidence intervals, effect sizes, degrees of freedom and P value noted<br>*Give P values as exact values whenever suitable.*
☒	☐	For Bayesian analysis, information on the choice of priors and Markov chain Monte Carlo settings
☐	☒	For hierarchical and complex designs, identification of the appropriate level for tests and full reporting of outcomes
☐	☒	Estimates of effect sizes (e.g. Cohen's d, Pearson's r), indicating how they were calculated

Our web collection on statistics for biologists contains articles on many of the points above.

Software and code

Policy information about availability of computer code

Data collection	Flow cytometry data were acquired with LRSFortessa flow cytometer and FACSDiva software v8.0.1 (BD Biosciences, Franklin Lakes, New Jersey, USA).<br>Echocardiographic data were acquired with Vevo 2100 System echocardiograph (Visual Sonics).<br>Catecholamines were quantified with HPLC system (LC20AT-Shimadzu).<br>Quantitative PCR data were collected with StepOnePlus system (Applied Biosystems).<br>Western blot images were acquired with ImageQuant LAS 500 (GE Healthcare Life Sciences).
Data analysis	All statistical analyses were performed with GraphPad Prism v 8.0.1<br>Flow cytometry data were analysed with FlowJo Software v 10.0.7<br>Heat maps were generated at: http://www.heatmapper.ca/ - Sasha Babicki, David Arndt, Ana Marcu, Yongjie Liang, Jason R. Grant, Adam Maciejewski, and David S. Wishart. Heatmapper: web-enabled heat mapping for all. Nucleic Acids Res. 2016. doi:10.1093/nar/gkw419

For manuscripts utilizing custom algorithms or software that are central to the research but not yet described in published literature, software must be made available to editors and reviewers. We strongly encourage code deposition in a community repository (e.g. GitHub). See the Nature Research guidelines for submitting code & software for further information.

Data

Policy information about availability of data

All manuscripts must include a data availability statement. This statement should provide the following information, where applicable:

- Accession codes, unique identifiers, or web links for publicly available datasets
- A list of figures that have associated raw data
- A description of any restrictions on data availability

All data supporting the results reported here are available upon reasonable request to the corresponding author. The source data for all the figures are included in this manuscript as Source Data file.

Field-specific reporting

Please select the one below that is the best fit for your research. If you are not sure, read the appropriate sections before making your selection.

- [x] Life sciences
- [ ] Behavioural & social sciences
- [ ] Ecological, evolutionary & environmental sciences

For a reference copy of the document with all sections, see nature.com/documents/nr-reporting-summary-flat.pdf

Life sciences study design

All studies must disclose on these points even when the disclosure is negative.

Sample size	The sample size was based on previous studies from our laboratory and literature (Zoccal, et al., 2016; Palm, N.W. & Medzhitov, R. 2013; von Moltke, et al., 2012; Monteiro et al., 2013) considering an alpha and beta errors of 0.05 and 0.20, respectively.
Data exclusions	There were no data exclusions.
Replication	Most of the experiments were independently repeated 2-3 times. All attemps of replication were successful.<br>Some experiments were performed only once because of the high sample size required to perform it. Ethical committe constrains also limited the total number of animals to be used in the project. Therefore, experiments that were critical to confirm the underlying hypothesis were prioritized to be replicated. Experiment replication is described in figure legends.
Randomization	Animals from the same genetic background were allocated together in the same cage and randomly selected for each experimental group.
Blinding	All animal experiments were not blinded because the researcher who performs the experiments must be trainned for animal and venom manipulation and for the identification of the "human end point", in accodance with Animal Care and Use Committee of Pharmaceutical Sciences and Medical Schools of Ribeirão Preto - USP.

Reporting for specific materials, systems and methods

We require information from authors about some types of materials, experimental systems and methods used in many studies. Here, indicate whether each material, system or method listed is relevant to your study. If you are not sure if a list item applies to your research, read the appropriate section before selecting a response.

Materials & experimental systems

n/a	Involved in the study	
☐	☒	Antibodies
☒	☐	Eukaryotic cell lines
☒	☐	Palaeontology and archaeology
☐	☒	Animals and other organisms
☒	☐	Human research participants
☒	☐	Clinical data
☒	☐	Dual use research of concern

Methods

n/a	Involved in the study	
☒	☐	ChIP-seq
☐	☒	Flow cytometry
☒	☐	MRI-based neuroimaging

Antibodies

Antibodies used	PKA-γ (clone: EP2647Y) (1:6000) (Abcam)<br>PKA (phospho T197) (clone: EP2606Y) (1:6000) (Abcam)<br>CD11b-BV510 (1:100) (clone: M1/70) (BD Biosciences)<br>CD45-APCCy7 (clone: 30-F11) (1:100) (BD Biosciences)<br>Ly6G-FITC (clone: 1A8) (1:100) (BD Biosciences)<br>MHC-II-APC (clone: M5/144.15.2) (1:100) (BD Biosciences)

F4/80-PECy7 (clone: BM8) (1:100) (Thermo Fisher Scientific)
CD31-PECy7 (1:100) (clone: EPR17259) (Abcam)
PDGFR-α-PE (1:100) (clone: APA5) (Abcam)
CD14-BV510 (clone: rmC5-3) (1:100) (BD Biosciences)
TLR4 (CD284)-BV421 (clone: MTS510) (1:100) (BD Biosciences)
TLR2- (CD282)-Alexa Fluor 647 (clone: 6C2) (1:100) (BD Biosciences)
All antibodies were tested using CompBeads (BD Biosciences) and are routinely applied in experiments and publications from our laboratory.
For references, see:
Souza, COS. Frontiers in Immunology, 2018; Zoccal, KF. Frontiers in Immunology, 2018 and Prado, MKB. Biomolecules, 2020.

Validation	For Western Blot analysis, validation was performed by manufacturer's. For PKA (phospho T197) (clone: EP2606Y) (1:6000) (Abcam), validation was performed by manufacturer using HeLa cell lysate treated with calyculin A. For PKA-γ (clone: EP2647Y) (1:6000) (Abcam), validation was performed by manufacturer using HeLa, MCF7, PC3 and A549 cell lysates. For Flow Cytometry, we defined the positive staining by limit of unstained cell control and negative control compensation particles set (BD Biosciences). For ELISA, we used recombinant proteins according to manufacturer's instructions.

Animals and other organisms

Policy information about studies involving animals; ARRIVE guidelines recommended for reporting animal research

Laboratory animals	Specie: Mice<br>Sex: Male<br>Strains and age:<br>C57Bl/6 - 6-8 weeks<br>Il1r1-/-: 6-8 weekes<br>Nlrp3-/-: 6-8 weeks<br>Asc-/-: 6-8 weeks<br>Casp1/11-/-: 6-8 weeks
Wild animals	In this study, we did not use wild animals.
Field-collected samples	In this study, we did not performe field-collection.
Ethics oversight	Maintenance and the experiments with mice were conducted in accordance with the Ethical Principles in Animal Research adopted by the National Council for the Control of Animal Experimentation (CONCEA), and approved by the Animal Care and Use Committee of the Faculdade de Ciências Farmacêuticas de Ribeirão Preto, (CEUA-FCFRP) at the Universidade de São Paulo (FCFRP-USP), Ribeirão Preto, São Paulo, Brazil (Process nº 16.1.1081.60.5) and by Animal Committee of FMRP-USP (002/2018-1)

Note that full information on the approval of the study protocol must also be provided in the manuscript.

Flow Cytometry

Plots

Confirm that:

- [x] The axis labels state the marker and fluorochrome used (e.g. CD4-FITC).
- [x] The axis scales are clearly visible. Include numbers along axes only for bottom left plot of group (a 'group' is an analysis of identical markers).
- [x] All plots are contour plots with outliers or pseudocolor plots.
- [x] A numerical value for number of cells or percentage (with statistics) is provided.

Methodology

Sample preparation	Heart cells suspensions were obtained after tissue digestion at 37∘C for 60 min in 1 ml/heart digestion buffer (RPMI 1640, Liberase LT, Roche, Basel, Switzerland, and DNase 0.5 mg ml-1, Sigma Aldrich, St. Louis, Missouri, USA). The tissue fragments were passed through a cell strainer with 100-μm-pore size (BD Biosciences, Franklin Lakes, New Jersey, USA). Next, the red blood cells were lysed, and the remaining cells were washed in PBS, centrifuged, and resuspended in RPMI 1640 containing 5% FBS. Suspensions of 1 × 106 cells from heart tissue were used in further analysis. The following antibodies were used: CD11b (clone: M1/70); CD45 (clone: 30-F11); Ly6G (clone: 1A8); MHC-II (clone: M5/144.15.2); F4/80 (clone: BM8); CD31 (clone: EPR17259); PDGFR-α (clone: APA5); CD14 (clone: rmC5-3); TLR4 (clone: MTS510); TLR2 (clone: 6C2).<br>The gates were defined using the Negative Control Compensation Particles Set (BD Bioscience), as well unstained cell control.
Instrument	Data acquisition was performed using a LRSFortessa flow cytometer and FACSDiva software v8.0.1 (BD Biosciences, Franklin Lakes, New Jersey, USA)
Software	FlowJo software v.10.0.7 (Tree Star, Inc, Ashland, OR, USA)
Cell population abundance	100.000 events were acquired for all samples

Gating strategy

The gating strategies are demonstrated in supplementary material. Basically, we defined the positive staining by limit of unstained cell control and negative control compensation particles set (BD Biosciences). In next step, we applied the cytometric gating hierarchy for analysis of FSC-H/FSC-A, SSC-A/FSC-A followed by CD45+ Ly6G+ (neutrophil) and CD11b+ F4/80+ MHC-II+ (macrophages). Also, we use the gating hierarchy for analysis of FSC-H/FSC-A, SSC-A/FSC-A followed by CD31- PDGFR+ TLR2+ TLR4+ CD14+ (fibroblasts).

☒ Tick this box to confirm that a figure exemplifying the gating strategy is provided in the Supplementary Information.
